# New Heusler compounds in Ni-Mn-In and Ni-Mn-Sn alloys

**DOI:** 10.1038/s41598-019-44179-2

**Published:** 2019-05-23

**Authors:** X.-Z. Li, W.-Y. Zhang, S. Valloppilly, D. J. Sellmyer

**Affiliations:** 1 Nebraska Center for Materials and Nanoscience, University of Nebraska, Lincoln, NE 68588 USA; 2 Department of Physics and Astronomy, University of Nebraska, Lincoln, NE 68588 USA

**Keywords:** Magnetic properties and materials, Electronic properties and materials

## Abstract

Rapidly quenched ternary Ni-Mn-T (T = In, Sn) alloys exhibit features associated with magnetic skyrmions, so that XRD, TEM, EDS, SAED and HREM investigations were carried out for structural characterization on the two alloy systems. In this paper, we report a new type of Mn-rich Heusler compound with a cubic unit cell, *a* = 0.9150 nm in Ni-Mn-In and *a* = 0.9051 nm in Ni-Mn-Sn, which coexist with a Ni-rich full-Heusler compound with defects, *a* = 0.6094 nm in Ni-Mn-In and *a* = 0.6034 nm in Ni-Mn-Sn. A further analysis of the experimental results reveals a close structural relationship between these two compounds.

## Introduction

Heusler compounds are intermetallics with intriguing magnetic properties^1–3^. The term derives from the name of the German mining engineer and chemist, Friedrich Heusler, who studied such compounds in 1903^4^. A typical compound contains two parts copper, one part manganese, and one part aluminum, i.e., Cu_2_MnAl. Surprisingly, the compound is ferromagnetic, even though all its elemental shows zero net magnetic moment by themselves. In 1934, Bradley and Rogers showed that the room-temperature ferromagnetic phase of Cu_2_MnAl was a fully ordered structure of the L2_1_ type^5^. This has a primitive cubic lattice of copper atoms with alternate cells body-centered by manganese and aluminum. The lattice parameter is *a* = 0.595 nm. Variant structures of Heusler compounds have been found since then. A vast collection of more than 1500 compounds are today known as Heusler and half-Heusler (or semi-Heusler) compounds.

**Figure 1:**
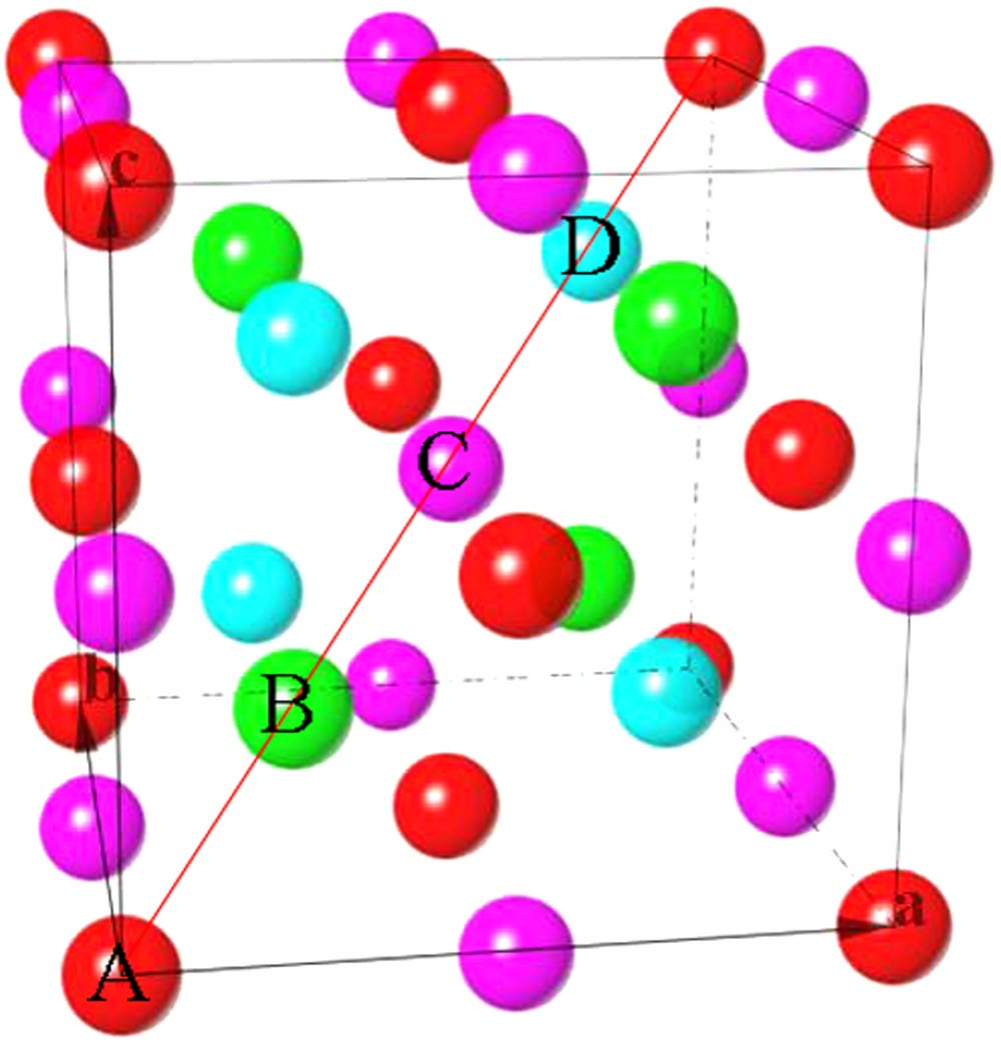
Structural prototypes of the Heulser compounds. X or X′ in red and pink, Y in green, and Z in blue.

The chemical compositions and atomic positions of the ordered and disordered Heusler compounds are summarized in Table 1 and shown in Fig. 1. Full-Heusler compounds crystallize in the L2_1_ structure (Cu_2_MnAl prototype, space group *Fm-*3*m*) and have a stoichiometric composition of X_2_YZ, where X and Y are transition metal elements, and Z is a group III, IV or V element^2,5^. If two X sites are occupied by X and X′, then a quaternary phase has the Y structure (LiMgPdSb prototype, space group *F-*43*m*). If X′ site is vacant in the Y structure, the half-Heusler compound in the C1_b_ structure (prototype AgAsMg, space group *F-*43*m*) is obtained with a composition of XYZ. The site disorder leading to solid solution in the B2 (CsCl prototype, space group Pm-3m) and the A2 (bcc structure, space group Im3m) structures is represented by Y/Z and X/Y/Z in Table 1, respectively. Inverse Heusler compounds in the XA structure (prototype Hg_2_CuTi, space group F-4m3) differs from the full Heusler structure (L2_1_) by half the X atoms interchanging positions with the Y or Z atoms^6^. The stability of the XA structure relative to the L2_1_ structure may be an atomic-size effect (*d*-band contraction), the XA order being favorable for small Y or Z atoms. The other factor favorable to XA order is the Y or Z element more electronegative than X.

**Table 1:** Summary on the structural prototypes of Heusler compounds.

Structure type	Composition	A	B	C	D
Y (Quaternary)	(XX’)YZ	X	Y	X′	Z
L2_1_ (Full)	X_2_YZ	X	Y	X	Z
C1_b_ (Half)	XYZ	X	Y		Z
XA (Inverse)	X_2_YZ	X	X	Y	Z
B2 (Partial order)	X_2_YZ	X	Y/Z	X	Y/Z
A2 (Full disorder)	X_2_YZ	X/Y/Z	X/Y/Z	X/Y/Z	X/Y/Z

Besides the cubic Heusler compounds listed above, tetragonal Heusler compounds are formed by expanding or contracting the unit cell in the *c*-direction^7^. The tetragonal alloys are normally described in terms of a different unit cell, characterized by a 45° rotation in the *a*-*b*-plane and *a*′ → *a*/√2. Each of the structures in Table 1 has its own tetragonal equivalent. A hexagonal Heusler analogue in Fe_2_MnGe alloy was reported in the crystal structure of DO_19_ with *a* = 0.522 nm and *c* = 0.424 nm^8^.

The initial purpose of this research project was to search and investigate skyrmions in Ni-Mn-T (T = Ga, In, Sn, Al) alloys. Magnetic skyrmions, in which the magnetic moments exhibit a characteristic swirling configuration, have attracted tremendous interest in condensed-matter physics because of their potential application in high-density information processing systems with high energy efficiency, spintronics, and microwave oscillators^9–11^. Our research results on the skyrmions were published separately^12^. In parallel research, structural characterization was carried out thoroughly in samples of rapidly quenched ribbons with a nominal composition of (Ni_0.5_Mn_0.5_)_65_T_35_ (T = Ga, Sn, In, and Al) alloys synthesized by using the melt-spinning technique. In this paper, we report a new type of Heusler compound in Mn-rich ternary alloy, which coexists with a full-Heusler compound with defects in composition of Ni_2-x_Mn_1+y_T_1−y_ (T = In, Sn) by using x-ray powder diffraction (XRD), transmission electron microscopy (TEM), energy-dispersive x-ray spectroscopy (EDS), selected-area electron diffraction (SAED) and high-resolution electron microscopy (HREM) techniques.

## Results and Discussion

Rapidly quenched ribbons with a nominal composition of (Ni_0.5_Mn_0.5_)_65_T_35_ (T = In or Sn) were examined by XRD, TEM, SAED and EDS and then further investigated by SAED tilt series and HREM imaging in details. The average chemical compositions of the compounds are listed in Table 2, which was obtained by measurement of multiple samples and multiple areas in each sample by EDS analysis; typical experimental results are shown in the Supplemental Materials. The average composition is near Ni_43.7_Mn_34.3_In_22.0_ for the primary crystalline phase, which can be described as Ni_2−x_Mn_1+y_In_1−y_ where x = 0.483, y = 0.206.

**Table 2:** Average composition in the samples together with the compositions of the full- and half- Heusler compounds.

Compound	Ni (at. %)	Mn (at. %)	In (at. %)
Primary phase in this study	43.7	34.3	22.0
New phase in this study	22.7	48.1	29.2
Full Heusler (ideal)	50	25	25
Half Heusler (ideal)	33.33	33.33	33.33

**Figure 2:**
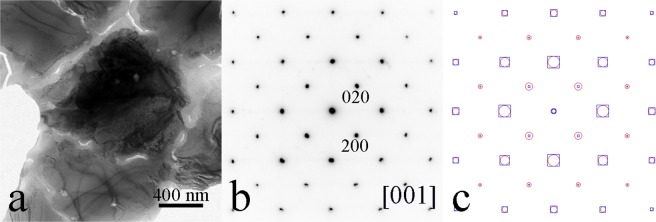
(**a**) A TEM image and (**b**) an SAED pattern of the full-Heusler compound with defects in composition of Ni_2−x_Mn_1+y_In_1−y_ where x = 0.483; y = 0.206. (**c**) Calculated patterns on the basis of the full-Heusler structure in blue squares and on the basis of the full-Heusler compound with defects in red circles.

Figure 2 shows: (a) a typical morphology of the Ni-Mn-In samples in a TEM image with a grain size about 1 μm; (b) a corresponding SAED pattern; and (c) calculated patterns on the basis of the full-Heusler compound in blue squares and on the basis of the full-Heusler with defects or the half-Heusler in red circles. The defects are partial vacancy in Ni atom sites and a mixture of Mn and In in In atom sites. The experimental atom radius is 0.140 nm for Mn element and 0.155 nm for In element. The Mn atoms may replace the In atoms in consideration of atomic size. The experimental SAED pattern matches better to the calculated pattern (red circles). The composition in EDS analysis and the simulation of experimental XRD pattern favor the interpretation of the full-Heusler with defects. Thus the grain under examination can be viewed as a full-Heusler compound with defects along [001] zone axis. The lattice parameter was refined with XRD analysis to obtain as *a* = 0.6094 nm.

**Figure 3:**
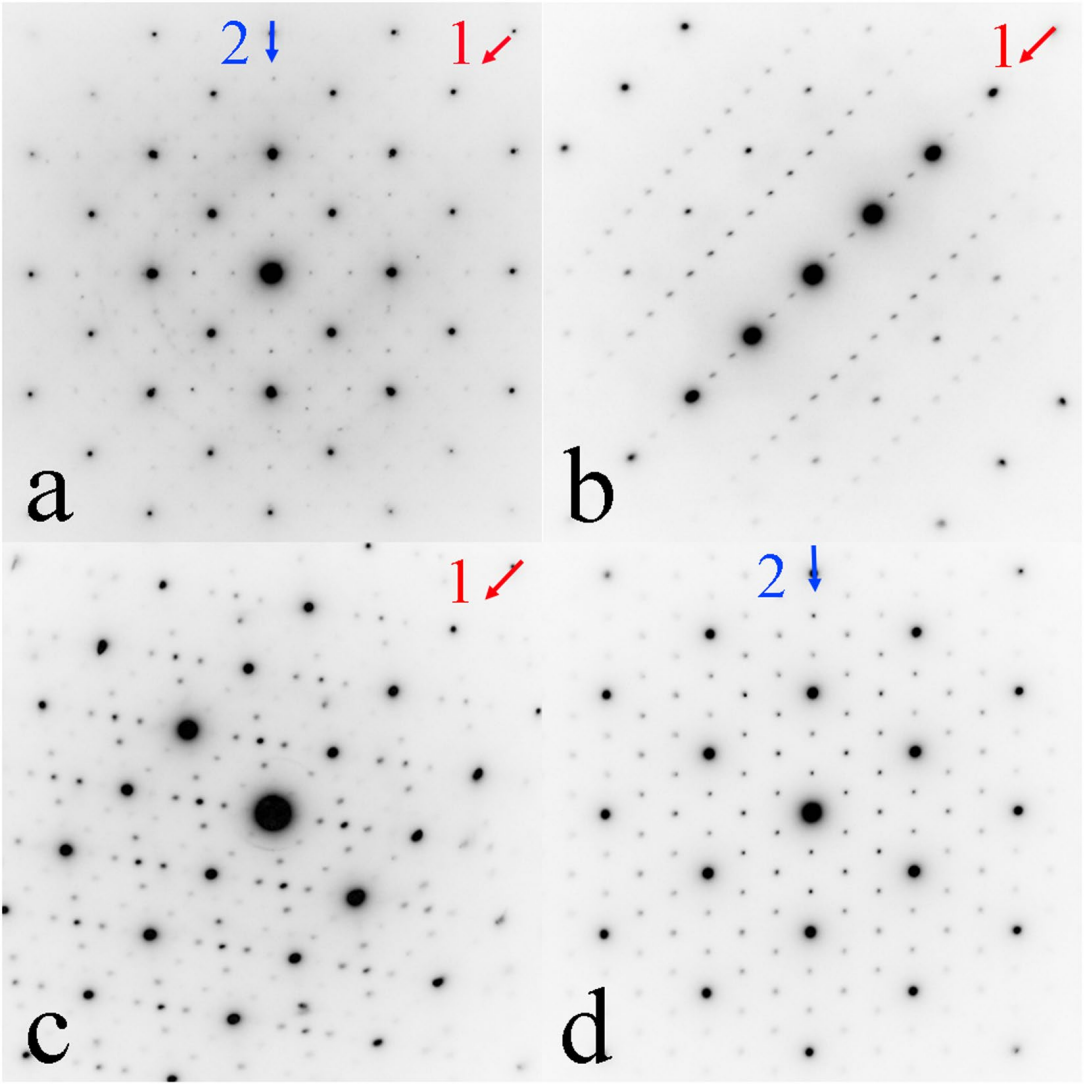
Experimental SAED patterns in the Ni-Mn-In sample. The patterns were taken from single grains in different orientation.

Besides the full-Heusler compound with defects as shown above, far more complicated SAED patterns were also observed as shown in Fig. 3(a–d). A detailed analysis shows that these SAED patterns can be interpreted as the composite patterns of the full-Heusler compound with defects and a new Heusler compound with cubic structure, *a* = 0.9150 nm. The average chemical composition of the new compound is also listed in Table 2. The new phase always coexists with the full-Heusler compound with defects, either inside the grain of primary phase or by the side of the grain of the primary phase. The same type of structure has also observed in Ni-Mn-Sn alloys as shown in Supplemental Materials. A tilt series of SAED patterns in Fig. 3 was obtained from single-crystalline grains by using a double tilting TEM holder. Figure 3(a–c) share the same row of reflections labeled by the arrow 1 and Fig. 3(a,d) share another row of reflections labeled by the arrow 2. The tilting angle from (a) to (b) is about 18.5°; from (a) to (c) is about 45°; from (a) to (d) is about 54.5°.

**Figure 4:**
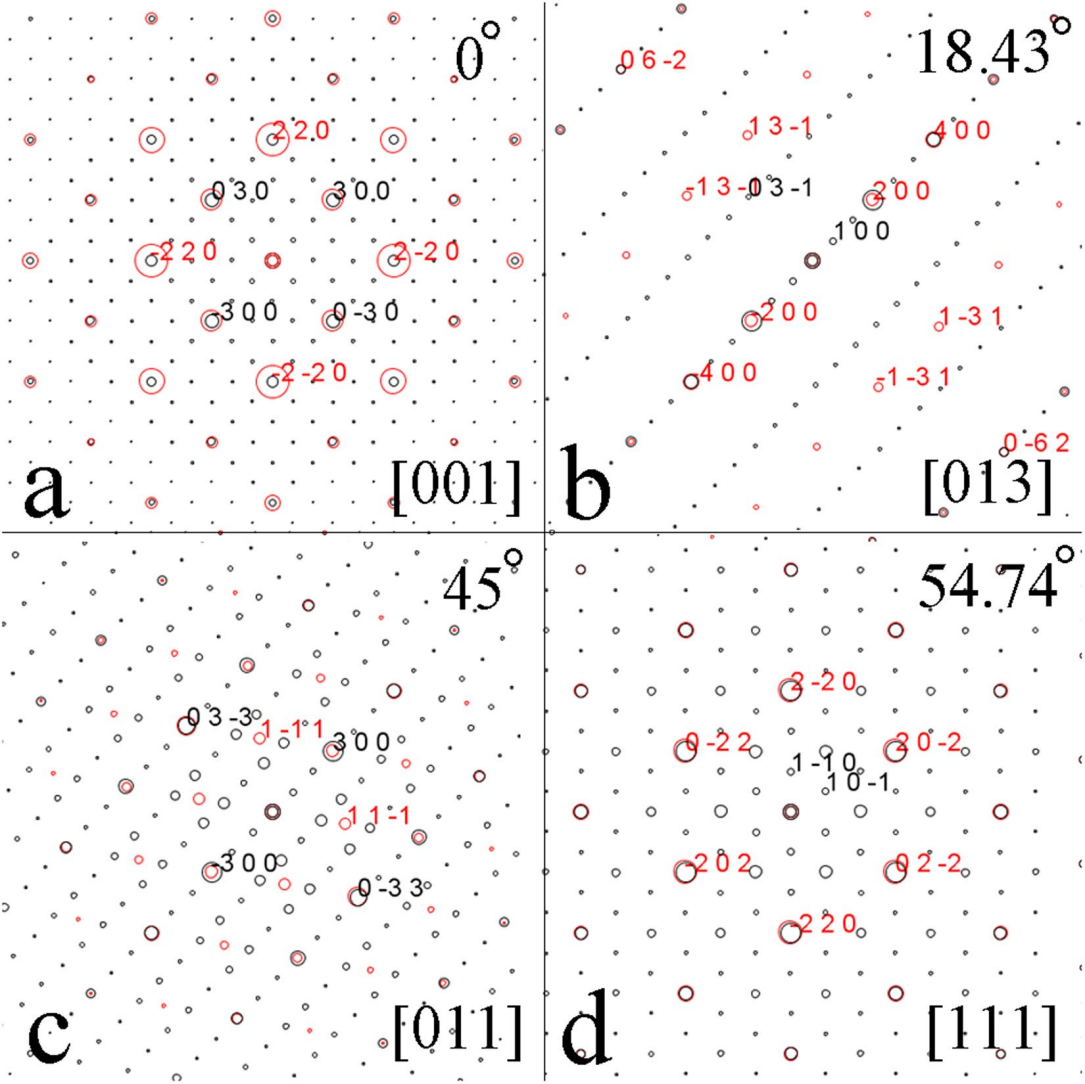
Simulation of a composite electron diffraction pattern from the full-Heusler compound with defects and the new Heusler compound. The positions of reflection spots in all calculated patterns match well with the corresponding SAED patterns in Fig. 3.

In order to confirm the above interpretation, a set of electron diffraction patterns is calculated on the basis of a composite of the full-Heusler compound with defects and the new Heusler compound, as shown in Fig. 4. The simulation was carried out on the basis of a simple model focusing on the positions of the reflections but not on the intensities in each pattern. The orientation of the two compounds have parallel edges for the two cubic cells. The reflections in black are from the new Heusler compound and the reflections in red are from the full-Heusler compound with defects. Although the reflections of the full-Heusler compound with defects totally coincide to the new Heusler compound in Fig. 4(a,d), the reflections of the full-Heusler compound with defects and the new Heusler compound can be distinguished in Fig. 4(b,c). They show that: (i) the tilt angles between all the patterns are in a good agreement to the experimental results; (ii) the positions of the reflections in the composite patterns are in a perfect agreement with the experimental results.

**Figure 5:**
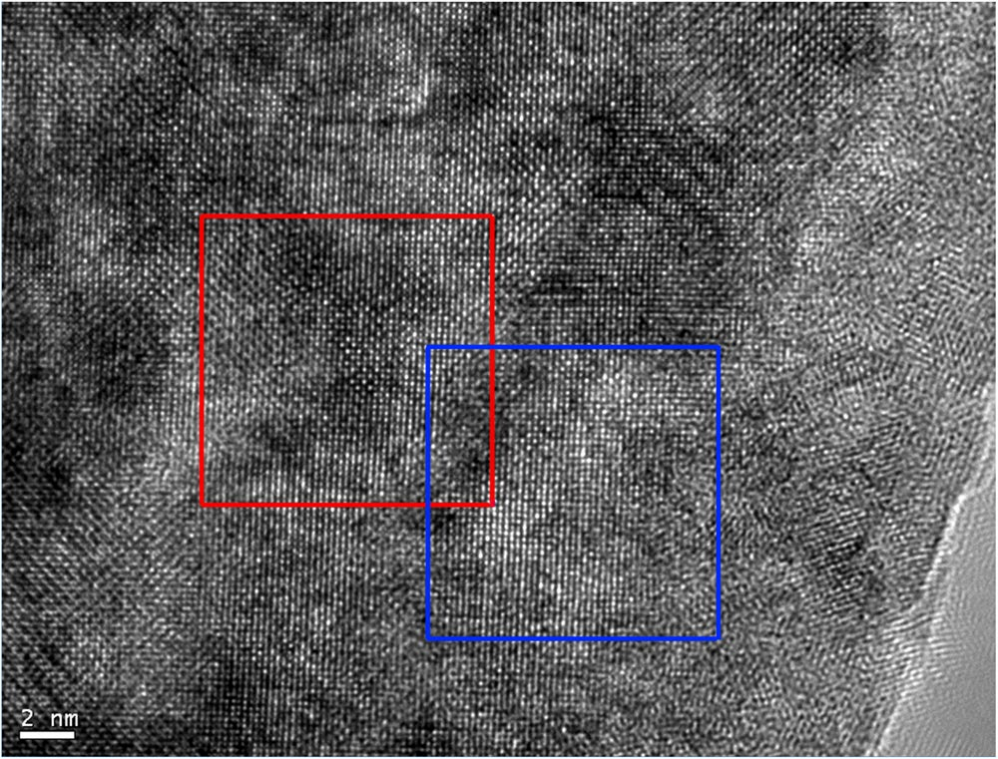
HREM images taken from an area of the Ni-Mn-In samples with an SAED pattern identical to the one in Fig. 3(a). Two areas are marked in squares for further analysis.

**Figure 6:**
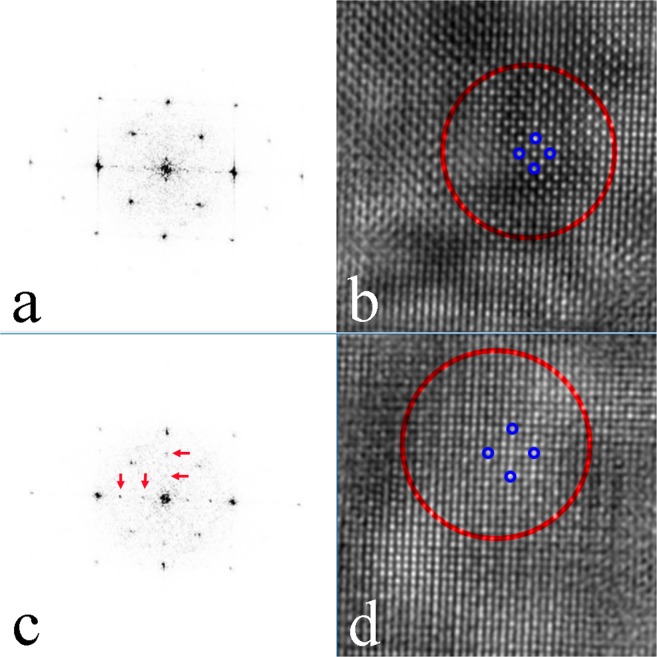
(**a**) FFT spectrum and (**b**) filtered image from the square area in red in Fig. 5. (**c**) FFT spectrum and (**d**) filtered image from the square area in blue in Fig. 5.

HREM experiments were carried out to further investigate the structural relationship between the new Heusler compound and the full-Heusler compound with defects. Figure 5 shows a HREM image taken on an area of the Ni-Mn-In samples, in which its SAED pattern is identical to the one in Fig. 3(a). Two square areas marked in Fig. 5 were selected for Fast Fourier Transformation (FFT) and then inverse FFT to get filtered images, as shown in Fig. 6. The FFT spectrum in Fig. 6(a) is similar to the SAED pattern of the full-Heusler compound with defects and in Fig. 6(b) is the filtered image which shows a domain of the full-Heusler compound with defects. The FFT spectrum in Fig. 6(c) has more fine spots than the FFT spectrum in Fig. 6(a) as marked with arrows and the filtered image in Fig. 6(d) shows the structural image which is different from the filtered image in Fig. 6(b). The results confirm this area is a domain of a new structure. Both a primitive lattice with an edge length of 0.9150/√2 nm or a centered square with an edge length of 0.9150 nm can be chosen to describe the structure projection of the new Heusler compound. The primitive lattice consists 9 basic squares on the filtered HREM image and the centered square lattice is marked in Fig. 6(d). In combination of the analysis of the XRD diffractogram and SAED patterns, the unit cell of the new Heusler compound in Ni-Mn-In should be a cubic structure with *a* = 0.9150 nm.

The composition of the Mn-rich ternary compound and the relatively large cubic lattice parameter remind us of the InMn_3_ phase^13,14^, which has a space group of *P*-43m and *a* = 0.942 nm. However, the calculated electron diffraction pattern of the [001] zone axis and the experimental electron nanodiffraction pattern of the new phase do not match each other. We concludes that the InMn_3_ is not a prototype of the new intermetallic compound.

**Figure 7:**
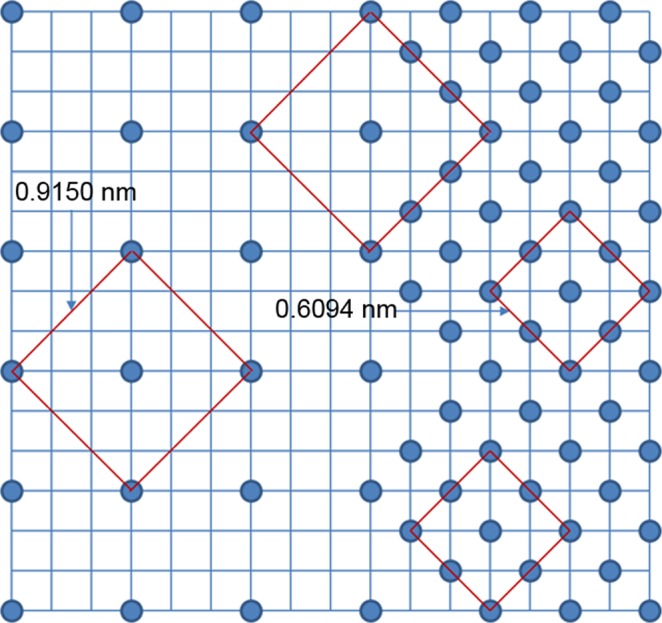
A schematic drawing of the relationship between the full-Heusler compound with defects and the new Heusler compound in Ni-Mn-In alloy system.

Figure 7 shows a schematic drawing of the structural relationship between the full-Heusler compound with defects and the new Heusler compound in the Ni-Mn-T (T = In and Sn) alloy systems. The two compounds grow together and have perfect grain boundaries at atomic level. There must be a close structural relationship between them. More experimental data on the new Heusler compound are required for constructing a reliable crystal structure model, and this will be a further task in our research.

## Conclusions

A new type of Mn-rich Heusler compound with a cubic structure, *a* = 0.9150 nm in Ni-Mn-In and and *a* = 0.9051 nm in Ni-Mn-Sn, has been found, which coexists with a full-Heusler compound with defects, *a* = 0.6094 nm in Ni-Mn-In and *a* = 0.6034 nm in Ni-Mn-Sn, respectively. The investigation shows that the new Heusler compound and the full-Heusler compound with defects have a close structural relationship and they coexist with perfect grain boundaries at the atomic level.

## Methods

Samples with a nominal composition of (Ni_0.5_Mn_0.5_)_65_T_35_ (T = In or Sn) in form of ribbons were prepared by arc melting, rapid quenching and annealing methods. First, the ingots were prepared by arc melting high-purity (99.95%) constituent elements in an argon atmosphere. In order to compensate for Mn loss, an extra 3% Mn by weight was added during arc melting. The crystalline ribbon samples were obtained by rapidly quenching the induction-melted materials onto the surface of a copper wheel rotating at 15 m s^−1^ in the melt-spinner chamber. These ribbons are about 2 mm wide and 50 *μ*m thick. In order to obtain the thermal equilibrium state of the samples, selected samples were further annealed at 500 °C for 2 h in a tubular furnace pumped to a base pressure of about 10^−7^ Torr.

The ribbons were first given a preliminary analysis with x-ray diffraction on crushed powder. It shows that all strong peaks can be indexed to the full-Heusler compound with defects. The peaks from the new phase are not easily identified due to i) the coincidence of the lattice parameters of the two phases, ii) the quantity of the new phase is much less than the primary phase and iii) the XRD data was collected as a fast measurement in the preliminary check. The XRD experiment was later repeated with a more fine powder and a long-time measurement. The peaks from the new phase can be identified (see Supplemental Materials). One peak at 2θ ≈ 33° cannot be indexed to either of the primary and the new phases, which indicates the existence of other minor crystalline phase in the sample. The lattice parameters of the primary and the new phases were refined from the XRD analysis and this confirmed their structural relation which was established in the SAED study.

The ribbon samples were cut into pieces approximately 2 mm long and mechanically ground and polished to approximately 30 mm or thinner. The samples were then mounted onto 3 mm diameter TEM Cu rings and milled to electron transparency using a Gatan PIPS II under the conditions of Ar ion-beam a 4 kV, with an angle starting at 10° and then decreasing to 6°. The SAED and HREM experiments were carried out on a Thermo Fisher Scientific (former FEI) Tecnai Osiris microscope equipped with a Gatan Orius CCD camera. The microscope operates at 200 kV and are equipped with a double-tilt TEM holder. Crystalline phases were first examined in SAED and EDS experiments. The EDS data were collected with the ChemiSTEM system on the Osiris microscope and analyzed with Bruker’s ESPRIT software. The tilt series of experimental SAED patterns were analyzed using SAED3^15^ and SPICA^16^ software. The experimental HRTEM images were processed by EMIPA software^17^.

## Supplementary information

Supplemental Materials
