# Supplementary material for: New Heusler compounds in Ni-Mn-In and Ni-Mn-Sn alloys
Source: Sci Rep. 2019 May 23;9:7762. doi: 10.1038/s41598-019-44179-2 (PMC6533245; doi:10.1038/s41598-019-44179-2)
Supplement: Supplementary file 1 — Supplemental Materials [file 41598_2019_44179_MOESM1_ESM.pdf]

Scientific Reports (Physical Sciences)
Reference #: SREP-18-48782

New Heusler compounds in Ni-Mn-In and Ni-Mn-Sn alloys

X.-Z. Li[1]*, W.-Y. Zhang[1,2], S. Valloppilly[1] and D.J. Sellmyer[1,2]

[1]*Nebraska Center for Materials and Nanoscience, University of Nebraska, Lincoln, NE 68588, USA*
[2]*Department of Physics and Astronomy, University of Nebraska, Lincoln, NE 68588, USA*

*Correspondence and requests for materials should be addressed to X.-Z. L. (email: xzli@unl.edu)

Supplemental Materials

Primary phase

Spectrum: 1355

El	AN	Series	unn. C [wt.%]	norm. C [wt.%]	Atom. C [at.%]	Error (1 Sigma) [wt.%]
Ni	28	K-series	36.85	36.85	43.78	1.13
Mn	25	K-series	26.97	26.97	34.24	0.83
In	49	K-series	36.18	36.18	21.98	1.11
		Total:	100.00	100.00	100.00	

a

New phase

Spectrum: 1603

El	AN	Series	unn. C [wt.%]	norm. C [wt.%]	Atom. C [at.%]	Error (1 Sigma) [wt.%]
Mn	25	K-series	36.07	36.07	47.98	1.12
Ni	28	K-series	18.63	18.63	23.19	0.60
In	49	L-series	45.30	45.30	28.83	[illegible]56
		Total:	100.00	100.00	100.00	

b

SM1. EDS analysis results on the primary phase and the new phase in Ni-Mn-In alloys

$$Ni_{43.7}Mn_{34.3}In_{22.0} = Ni_{2-x}Mn_{1+y}In_{1-y}$$

$$\frac{2-x}{4-x} = 43.7\%; \ \frac{1+y}{4-x} = 34.3\%; \ \frac{1-y}{4-x} = 22.0\%$$

$$x=0.483; \ y=0.206$$

SM2. Full Heusler phase in composition of $Ni_{43.7}Mn_{34.3}In_{22.0}$ with defects, partial vacancy in Ni atom sites and a mixture of Mn and In in In atom sites.

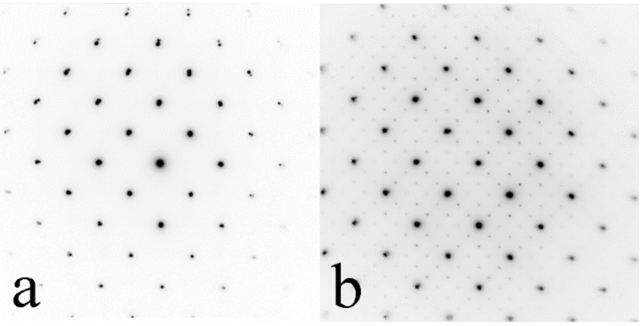

SM3. (a) SAED pattern of full-Heusler compound with defects, cubic, a = 0.6034 nm in Ni-Mn-Sn alloy and (b) a composite SAED pattern of a full-Heusler compound with defects and a new Heusler compound, cubic, a = 0.9051 in Ni-Mn-Sn alloys, Indices for two patterns are same as Figure 2(b) and Figure 4(a), respectively.

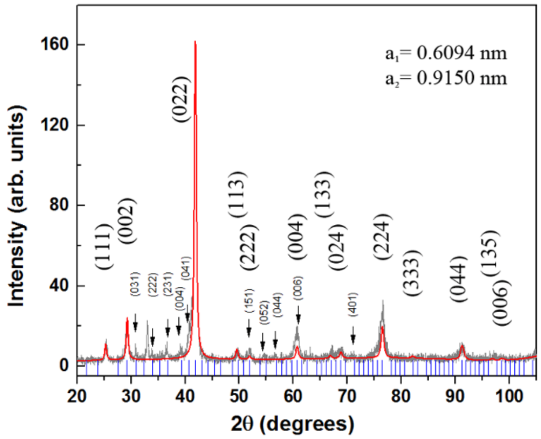

SM4. XRD diffractogram of a powder sample prepared from rapidly quenched ribbons of Ni-Mn-In alloy. Experimental data is shown in black and simulated data in red. Indices in large font size were based on a full-Hesuler with defects, space group: F-43m and a = 0.6094 nm. Indices in small font size were based on the new phase with a = 0.9150 nm.
